# When monopolar electrosurgery is not an option – successful bipolar endoscopic submucosal dissection for early gastric cancer

**DOI:** 10.1055/a-2928-8240

**Published:** 2026-08-14

**Authors:** Alda Andrade, Ana Rita Ribeiro, Jorge Bezerra, Helena Ribeiro, João Sousa, Luís Correia Gomes, Luís Lopes

**Affiliations:** 1 Gastroenterology Department 208305 ULS Alto MinhoViana do CasteloPortugal; 2 ICVS/3B’s – PT Government Associate LaboratoryBraga/GuimarãesPortugal; 3 Life and Health Sciences Research Institute (ICVS) 224744 University of Minho School of MedicineBragaPortugal

Endoscopic submucosal dissection (ESD) is an established method for en bloc resection of early gastric cancer. While monopolar knives are standard, monopolar current disperses energy toward a distant return electrode, increasing thermal spread and posing risks of deep tissue injury and electromagnetic interference in patients with implanted electronic devices such as cochlear implants. 1​ Bipolar systems, which confine current locally, reduce lateral thermal spread and may therefore be safer in selected patients. We report a gastric ESD performed using a bipolar knife in a patient with a cochlear implant.

**Fig. 1:**
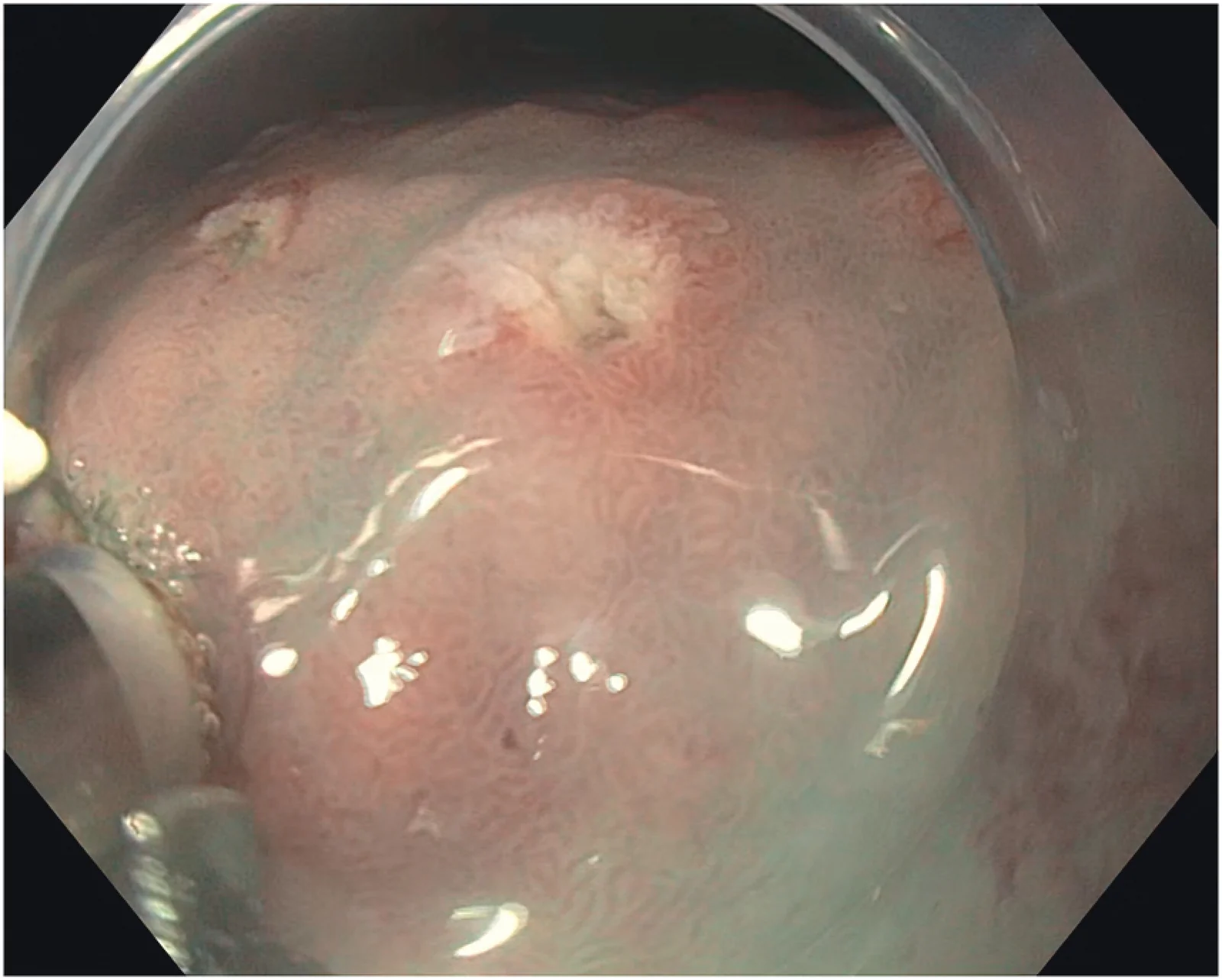
Initial mucosal incision using a bipolar knife. The return electrode is integrated into the transparent distal hood, confining electrical current between the knife tip and the hood-mounted electrode.

A 72-year-old man was referred for the endoscopic resection of a 15-mm flat lesion on the anterior wall of the mid-gastric body, with biopsies confirming adenocarcinoma. The patient had a cochlear implant, making monopolar electrosurgery unsuitable. Given the lesion’s morphology and the goal of curative en bloc resection, bipolar ESD was chosen as the safest and most effective approach. High-definition white-light and chromoendoscopy revealed a Paris 0-IIa lesion without deep invasion features. After submucosal injection, circumferential incision and dissection were performed using a bipolar needle-knife (B-knife), with hemostasis achieved using bipolar coagulation forceps ( Fig. 1 and Video 1 ). The B-knife, developed by Doi et al. with Zeon Medical in 2002, confines current to the needle tip, concentrating energy near the sheath pole and minimizing lateral thermal spread and deep muscle injury. 1​ Monopolar systems disperse current toward a distant electrode, increasing potential thermal damage and risks in patients with implanted devices. Although early bipolar knives had slower cutting performance, newer platforms provide efficiency comparable to monopolar knives. 2​ In this case, dissection proceeded with rapid, precise cutting, excellent field stability, and controlled coagulation. The procedure was completed in 180 minutes, yielding a 45×20 mm specimen without adverse events.

**Video 1** Bipolar endoscopic submucosal dissection of early gastric cancer in a patient with a cochlear implant using a hood-mounted return electrode.


En bloc R0 resection was achieved, with histopathology confirming a well-differentiated intramucosal adenocarcinoma with negative margins. As cochlear implants become more common, bipolar ESD is a valuable alternative for curative therapy in patients in whom monopolar current is contraindicated or undesirable, including those with pacemakers, implantable cardioverter-defibrillators, abandoned intracardiac leads, or neurostimulators.

Endoscopy_UCTN_Code_TTT_1AO_2AG_3AD
